# Atypical Presentation of Acute Mitral Regurgitation Secondary to Papillary Muscle Rupture

**DOI:** 10.7759/cureus.24744

**Published:** 2022-05-05

**Authors:** Kushal H Porwal, Mokshal H Porwal, Mohammed M Ibrahim, Hariharan Ramaswamykanive, Krishan Gupta, Manu Mathur, Seshasayee Narasimhan

**Affiliations:** 1 Internal Medicine, Manning Base Hospital, Taree, AUS; 2 Cardiology, Medical College of Wisconsin, Wauwatosa, USA; 3 Intensive Care Medicine, Manning Base Hospital, Taree, AUS; 4 Cardiothoracic Surgery, Royal North Shore Hospital, Sydney, AUS; 5 Cardiology, Internal Medicine, Manning Base Hospital, Taree, AUS; 6 Conjoint Senior, University of Newcastle, Callaghan, AUS; 7 Adjunct Senior Lecturer, University of New England, Armidale, AUS

**Keywords:** acute mitral regurgitation, papillary muscle rupture, flail mitral leaflet, mitral regurgitation, cardiogenic shock

## Abstract

Acute mitral regurgitation (MR) is a life-threatening condition presenting with severe decompensated heart failure due to sudden retrograde blood flow into the left atrium. The causes are broadly classified into ischemic and non-ischemic. Rapid and accurate diagnosis of acute MR and its potential causes is essential. This case uniquely highlights an atypical presentation of severe MR secondary to papillary muscle rupture without a known, identifiable cause. Therefore, suspicion of acute MR should be high if clinical symptoms are present, even without known risk factors, due to the high morbidity and mortality associated with delayed management.

## Introduction

Acute mitral regurgitation (MR) is a life-threatening condition presenting with severe decompensated heart failure due to sudden retrograde blood flow into the left atrium [1]. Patients with acute MR usually present with severe congestive heart failure (CHF) causing symptoms of pulmonary congestion, hypotension, and cardiogenic shock. Some common causes include ruptured chordae tendineae, myxomatous degeneration of the leaflet and chordae, and infective endocarditis. Additionally, papillary muscle rupture is a major cause of acute MR and is oftentimes fatal [2,3]. Therefore, optimal management warrants rapid diagnosis and surgical referral. We present a unique case of a 69-year-old female with an atypical presentation of severe MR secondary to unexplained papillary muscle rupture.

## Case presentation

A 69-year-old female presented with acute delirium in the setting of depression and bioprosthetic aortic valve replacement (AVR) with ascending aorta replacement (Bentall’s procedure) in October 2021 for severe aortic stenosis secondary to the bicuspid aortic valve, failed radiofrequency ablation for atrial fibrillation in June 2021 treated with rate-control and oral anticoagulation therapy, non-obstructive proximal and middle left anterior descending (LAD) and middle lateral circumflex (LCX) coronary artery disease with <25% stenosis noted on computed tomography (CT) coronary angiogram in October 2021, hypercholesteremia, and radio-contrast intolerance. Of note, an echocardiogram completed in March 2021 revealed a severely dilated left atrium with mild-to-moderate MR. Her current medications on admission included rivaroxaban 20 mg once daily, bisoprolol 5 mg once daily, digoxin 125 µg once daily, and rosuvastatin 10 mg once daily. She was prescribed olanzapine 7.5 mg by her family doctor prior to admission. She had no official diagnosis of dementia or other mental illnesses prior to admission.

Initial non-contrast CT was completed and showed no evidence of intracranial hemorrhage. This was followed by magnetic resonance imaging (MRI) of the brain which was performed for structural brain assessment. The study showed mild-to-moderate small-vessel ischemia, which was an incidental finding. A neurological examination showed no focal neurological findings. On advice from the neurologist, a CT cerebral angiogram was conducted, which was unremarkable.

**Figure 1:**
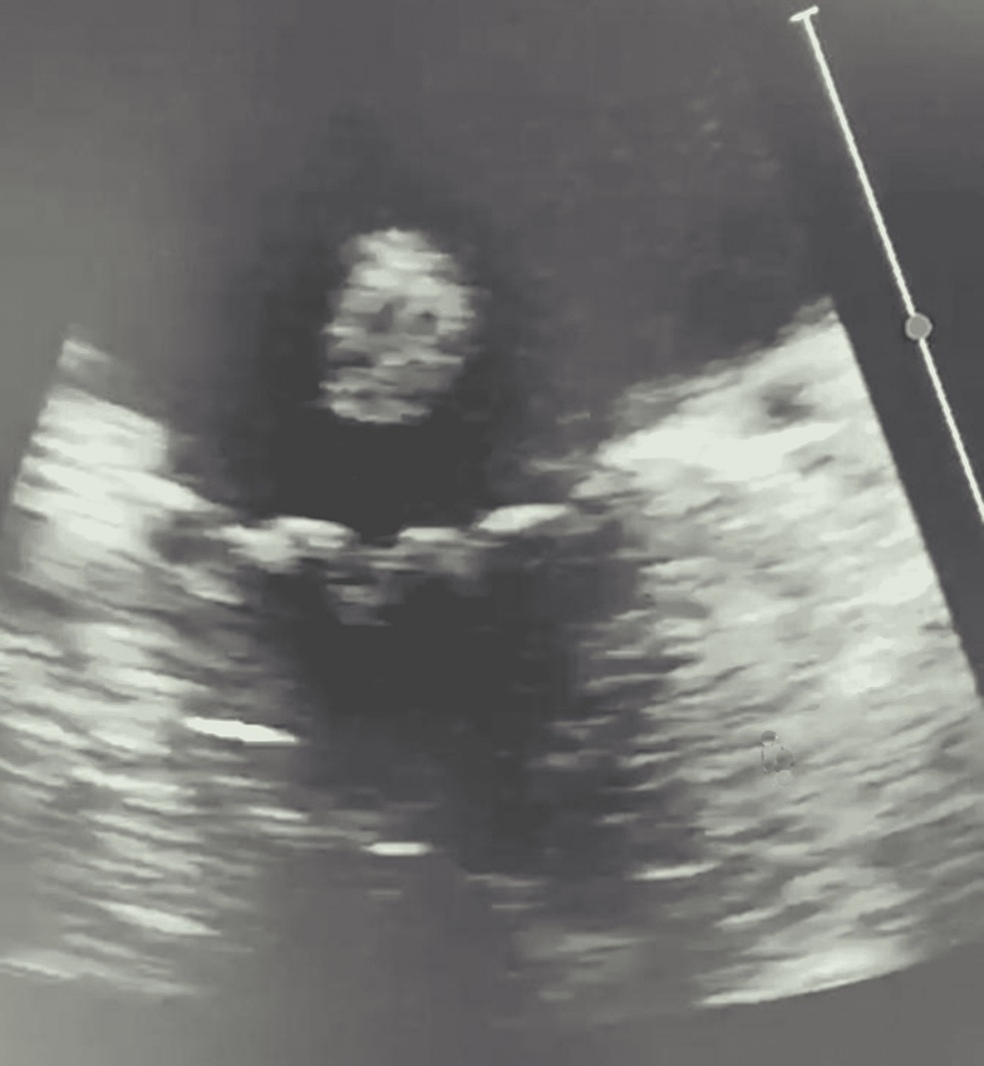
Transoesophageal echocardiogram demonstrating flail anterior mitral leaflet.

**Figure 2:**
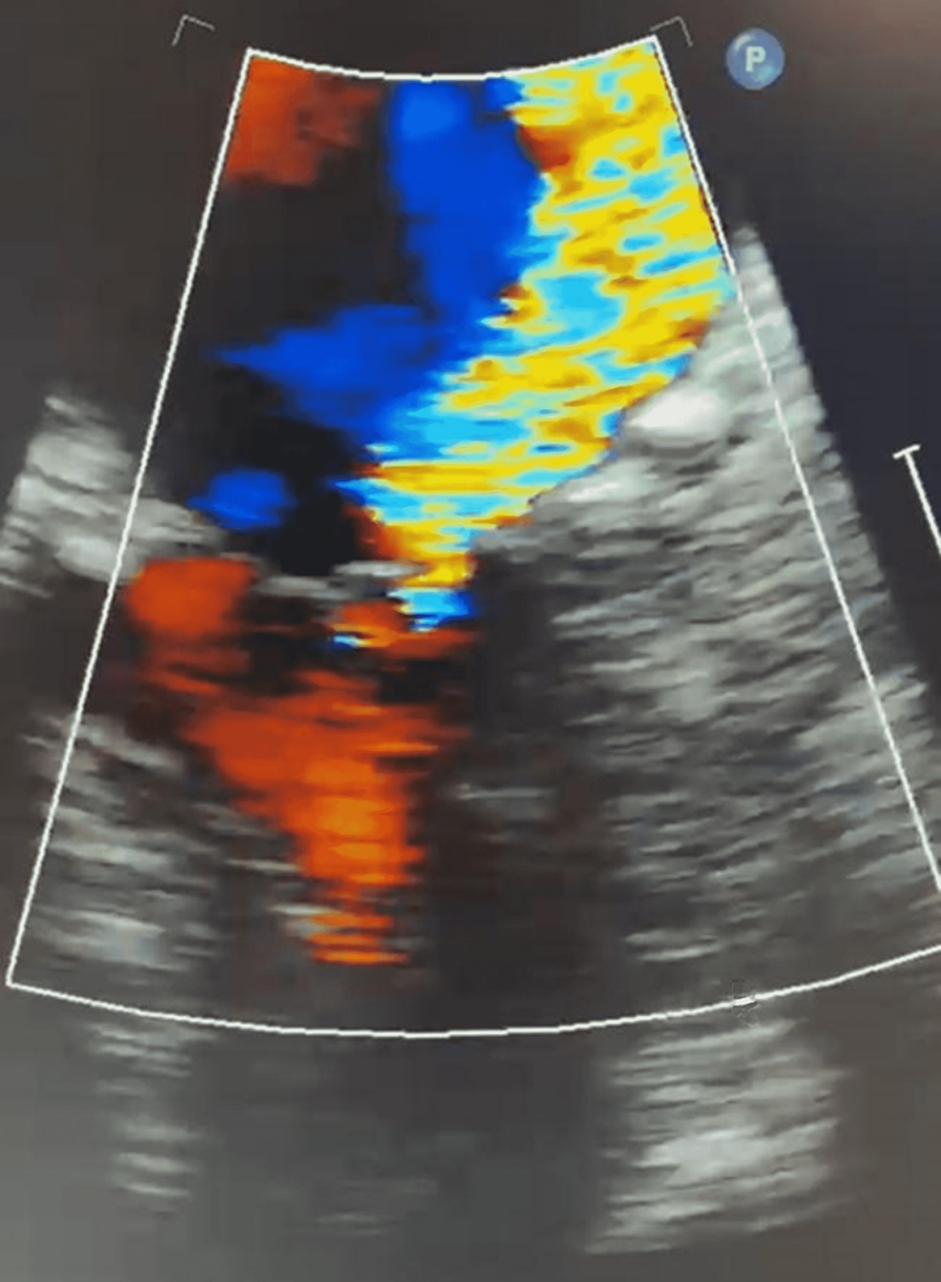
Close-up transoesophageal echocardiogram demonstrating posteriorly directed eccentric jet of severe mitral regurgitation.

**Figure 3:**
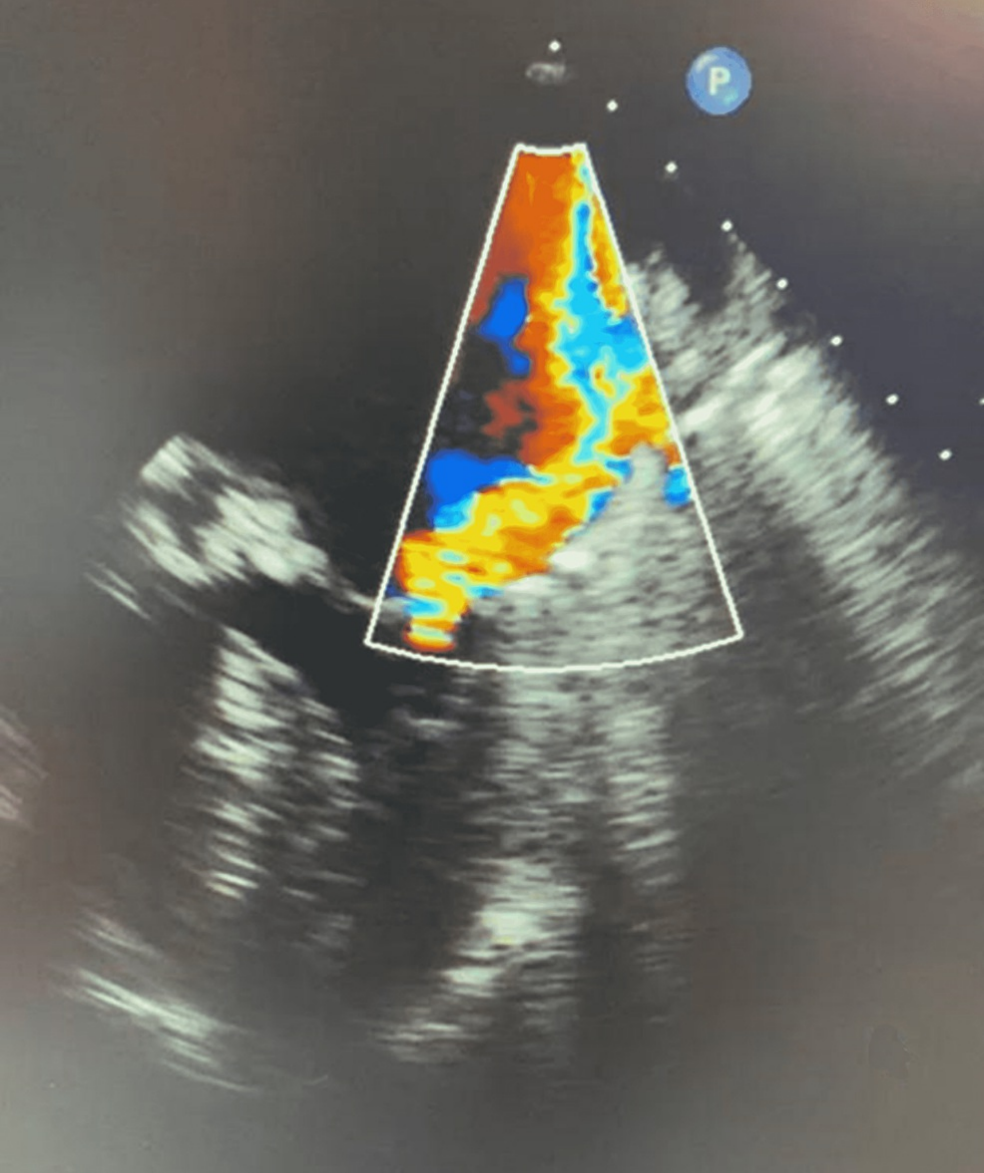
Transoesophageal echocardiogram demonstrating posteriorly directed eccentric jet of severe mitral regurgitation including flow reversal into the left lower pulmonary vein.

**Figure 4:**
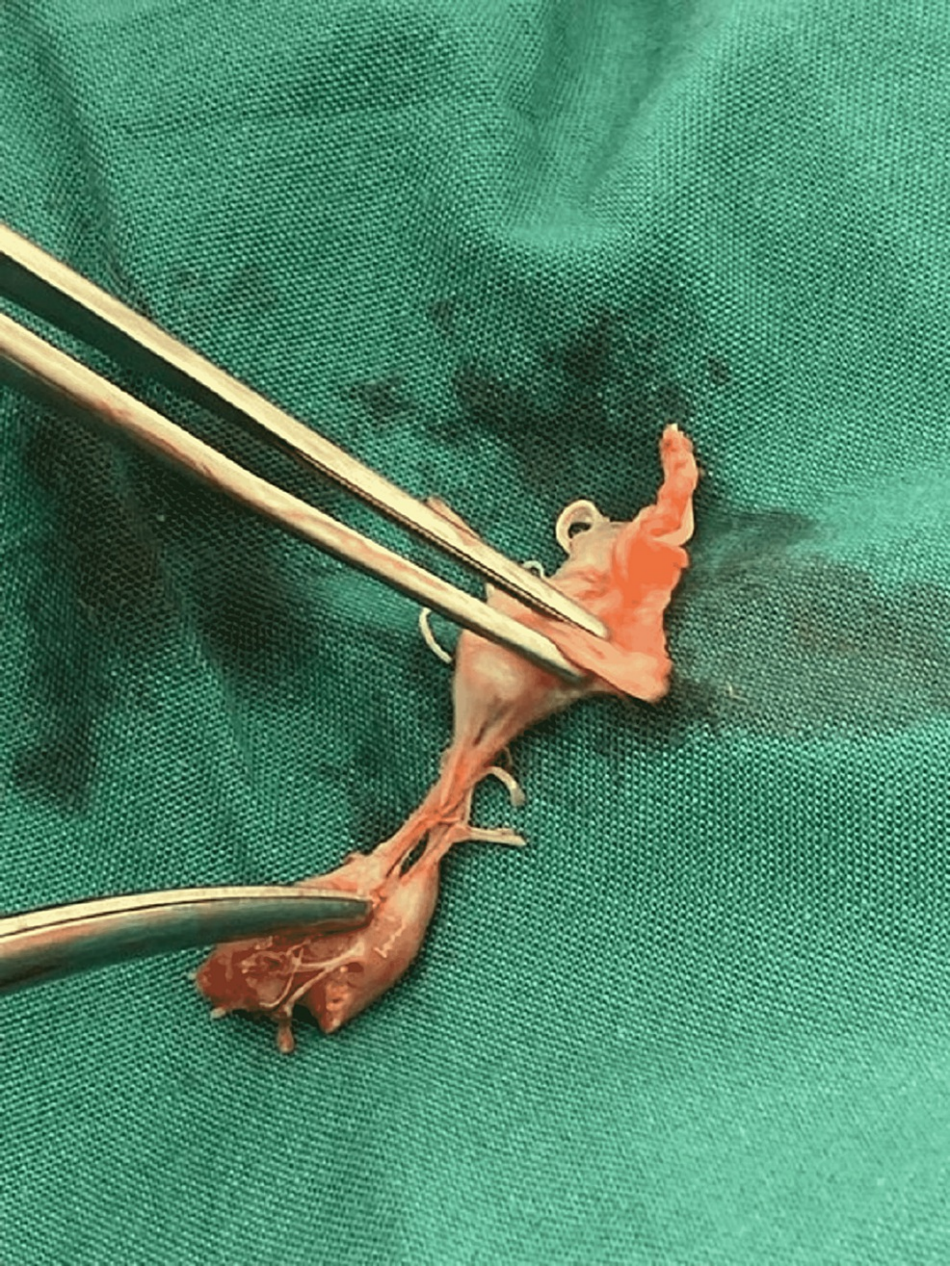
Ruptured papillary muscle with attached anterior mitral leaflet.

Approximately 15 minutes after administering intravenous contrast for the cerebral angiogram, the patient had a rapid response due to the sudden onset of hypoxia (53% SpO_2_ on room air) with severe hypotension (60/35 mmHg). No significant metabolic abnormalities were noted on admission, and there were no signs of alcohol intoxication or withdrawal. She deteriorated into acute pulmonary edema, which was confirmed on a chest X-ray. She was reviewed urgently by the Intensive Care Unit (ICU) team and transferred to the ICU for further stabilization. The patient was acutely delirious. In the ICU, her initial laboratory testing showed acute kidney injury, ischemic hepatitis, a rising troponin from 35,000 to 41,000 ng/L, and a pro-brain natriuretic peptide (BNP) of 21,000 ng/L. Her admission electrocardiogram to the ICU showed atrial fibrillation with a rapid ventricular rate and inferolateral ST-T changes. A bedside echocardiogram completed in the ICU showed an ejection fraction of approximately 30%. A summary of the patient’s relevant lab values and measurements is shown in Table 1. Given the hypotension, features of end-organ damage, and moderate-to-severe systolic dysfunction, intravenous (IV) dobutamine infusion was started. Blood cultures were analyzed and revealed positive findings for coagulase-negative staphylococci in one bottle. Given the presence of a bioprosthetic aortic valve, an urgent transesophageal echocardiogram (TOE) was conducted to rule out infective endocarditis. TOE was evident for flail anterior mitral valve leaflet (Figure 1) with a posteriorly directed eccentric jet of severe MR (Figures 2, 3). The patient was transferred to cardiothoracic for emergent mitral valve surgery. An urgent repeat invasive assessment confirmed mild coronary artery disease. The patient underwent a redo sternotomy with mitral valve replacement using a porcine bioprosthesis on day two of the transfer. The etiology of the severe MR was papillary muscle rupture, and the tissue appeared infarcted intraoperatively (Figure 4). Postoperatively, the patient was transferred to the ICU in stable condition with no bleeding. She continued to improve and was transferred to the rehabilitation ward to address her deconditioning prior to discharge.

**Table 1:** Laboratory values and measurements.

Measurement	Patient value	Reference
Troponin	35,000 to 41,000 ng/L (rising)	<0.04 ng/mL
Pro B-type natriuretic peptide	21,000 ng/L	<300 ng/L
Oxygen saturation on room air	53%	>95%
Ejection fraction	30%	50–75%

## Discussion

Acute MR usually presents as a cardiac emergency with the sudden onset and rapid progression of pulmonary edema, hypotension, and symptoms of cardiogenic shock [1]. As a result, acute MR requires urgent medical and surgical treatment. Compared to chronic MR, the hemodynamic changes in acute MR are more severe due to the lack of time for the left atrium and left ventricle to adapt to the high output and retrograde blood flow [4]. Therefore, effective forward flow is limited because the left ventricle is not dilated and a large fraction of ejected blood travels retrograde across the mitral valve. Despite a compensatory increase in heart rate, forward cardiac output falls, which can precipitate cardiogenic shock [1]. Additionally, a neurohumoral response to the reduction in cardiac output initiates. This causes an increase in vascular resistance, which exacerbates the regurgitation and leads to maladaptive cardiac remodeling, congestion, and increased morbidity and mortality [5].

There are several causes of MR, and a rapid and accurate diagnosis is imperative in the treatment course and prognosis. Broadly, the causes of MR are classified into ischemic and non-ischemic [6]. Ischemic MR can be caused by coronary disease and myocardial infarction which can cause papillary muscle displacement or rupture. Non-ischemic MR includes all other causes such as myxomatous valve disease, infective endocarditis, blunt chest trauma, rheumatic heart disease, spontaneous rupture, and dynamic left ventricular outflow obstruction. Additionally, iatrogenic causes of acute MR have been reported rarely in patients undergoing transcatheter aortic valve implantation [7] and removal of transaortic left ventricular assist devices [8] due to trauma to the leaflets or chords.

Treatment of acute and severe MR requires urgent surgical valve repair or replacement [9]. The role of medical therapy is limited to hemodynamic stabilization, diminishing progression, and reducing pulmonary congestion. In normotensive patients, this can be achieved using nitroprusside. However, in hypotensive patients, a combination of an inotropic agent such as dobutamine should be used due to severely reduced forward flow of blood [10]. Additionally, intra-aortic balloon pumping can reduce regurgitation and aortic impedance which acts to stabilize the patient while preparing for surgery [11].

In this case, our patient went into flash acute pulmonary edema in the setting of contrast intolerance and acute kidney injury immediately after the CT brain angiogram. This is not a known cause of papillary muscle rupture or acute MR. Additionally, her troponins reflected acute decompensated CHF. In the setting of unremarkable CT coronary angiogram in October 2021 and no chest pain, ischemic MR is less likely in this case. The diagnostic workup did not reveal any known etiology to explain her diagnosis of papillary muscle rupture and presentation of acute MR. Therefore, this is a unique case of an atypical presentation of severe MR secondary to papillary muscle rupture without an identifiable cause. Therefore, suspicion of acute MR should be high if the clinical scenario fits due to high morbidity and mortality.

## Conclusions

Acute MR is a life-threatening condition presenting with severe decompensated heart failure due to sudden retrograde blood flow into the left atrium. The causes are broadly classified into ischemic and non-ischemic. Rapid and accurate diagnosis of acute MR and its potential causes is essential. In this case, we highlight an atypical presentation of severe MR secondary to papillary muscle rupture without a known, identifiable cause. Therefore, if clinical symptoms are present, even in the absence of known risk factors, suspicion of acute MR should be high due to the high morbidity and mortality associated with delayed management.
